# Shunting Across a Latent Patent Foramen Ovale (PFO) in a Patient With Right Ventricular (RV) Infarction Improved With Impella

**DOI:** 10.7759/cureus.34302

**Published:** 2023-01-28

**Authors:** Sudarshan Gautam, Norbert Moskovits, Suvash Shrestha, Arjun Basnet, Armando Seitllari

**Affiliations:** 1 Internal Medicine, Maimonides Medical Center, Brooklyn, USA; 2 Cardiology, Maimonides Medical Center, Brooklyn, USA

**Keywords:** impella rp, refractory hypoxemia, right ventricular infarction, patent foramen ovale, right-to-left shunt

## Abstract

The right-to-left shunt (RTLS) through a latent patent foramen ovale (PFO) is a rare complication of right ventricle myocardial infarction (MI). Though a rare complication, the development of refractory hypoxemia after right ventricular MI should always alert clinicians to consider the possibility of shunting across PFO. Right-sided Impella (Impella RP) can be considered in such patients, which helps to decrease the elevated right heart pressure reducing the shunt, thereby providing a bridge to recovery.

## Introduction

Right ventricular infarction can lead to right ventricular failure with a rise in right ventricular and right atrial pressure (RAP). The elevated RAP can cause right-to-left shunting (RTLS) across a latent patent foramen ovale (PFO), bypassing the pulmonary circulation with eventual refractory hypoxemia [1]. Impella is a catheter-based temporary ventricular assist device that helps pump blood in ventricular failure [2]. Impella RP is the only Impella device approved for right heart circulatory support [3]. This device pumps blood from the inferior vena cava to the pulmonary artery. We present a case of a 63-year-old female admitted for a right ventricular infarct who developed refractory hypoxemia after the opening of latent PFO and improved with an Impella RP. This is the second reported case of Impella RP in refractory hypoxemia due to RTLS across PFO in the right ventricle infarct, to the author's knowledge [4].

## Case presentation

A woman in her early 60s with a past medical history of breast cancer in remission presented to the emergency department with chest pain for 3 h. Chest pain was central, sudden onset, severe intensity, crushing type, radiating to the epigastric area, associated with nausea and headache, and not relieved by rest. She denied fever, chills, shortness of breath, diaphoresis, vomiting, weakness, back pain, or lower extremity swelling. Her vitals in the emergency room were stable, and her physical examination findings were unremarkable. Electrocardiogram (EKG) showed sinus rhythm without ST-segment elevation and T wave changes. Her troponin trended from an initial 0.86 nanogram per milliliter (ng/mL) to 34 ng/mL over 4 h. She continued to have chest pain and was prompted to shift to the Cath lab for a left heart catheterization. 

**Figure 1:**
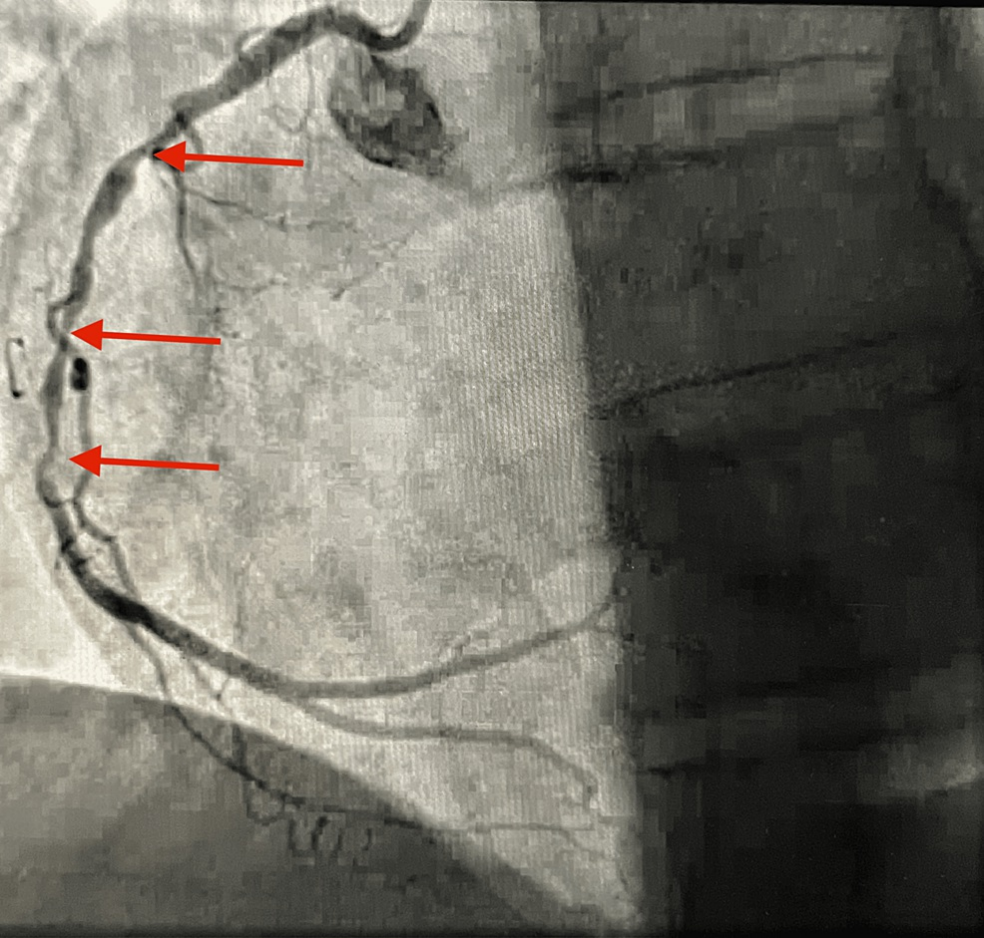
RCA showing diffuse disease (red arrow) with 80% mid-RCA occlusion. RCA, right coronary artery

Left heart catheterization showed triple vessel coronary artery disease with 80% occlusion in the mid right coronary artery (RCA) with diffuse disease (Figure 1). She underwent per-cutaneous intervention (PCI) of mid-RCA with balloon angioplasty without reflow, likely due to thrombus burden, followed by placement of two drug-eluting stents without successful reestablishment of flow. She received multiple rounds of intracoronary adenosine without improvement in blood flow (TIMI score 1). She became hemodynamically unstable with episodes of bradycardia and hypotension. The patient was intubated for airway protection. She received atropine and transvenous pacing via the right femoral vein for bradycardia. She became persistently hypotensive even with pacing, and an intra-aortic balloon pump (IABP) was placed for hemodynamic support. She was admitted to the critical care unit for further management. Echo showed global cardiomyopathy with 20% left ventricular ejection fraction (LVEF). Her oxygen requirement increased to 100% with an increase in positive end-expiratory pressure (PEEP). Nor-epinephrine and vasopressin drip started for pressure support. Chest imaging did not reveal any acute lung pathology, including pulmonary embolism.

Her vasopressors requirement went up, and dobutamine was added. Heparin drip was continued for IABP. Dual antiplatelets, aspirin, and ticagrelor were continued. Arterial blood gas showed worsening metabolic acidosis with peak lactic acid of 20 millimoles per liter (mmol/L). Right heart catheterization was done, which showed elevated RAP of 24 mmHg, pulmonary artery pressure (PAP) of 35/25 mmHg, and wedge pressure of 19 mmHg. Her pulmonary artery pressure pulsatility (PAPi) score was 0.4, indicating severe right ventricular dysfunction.

**Figure 2:**
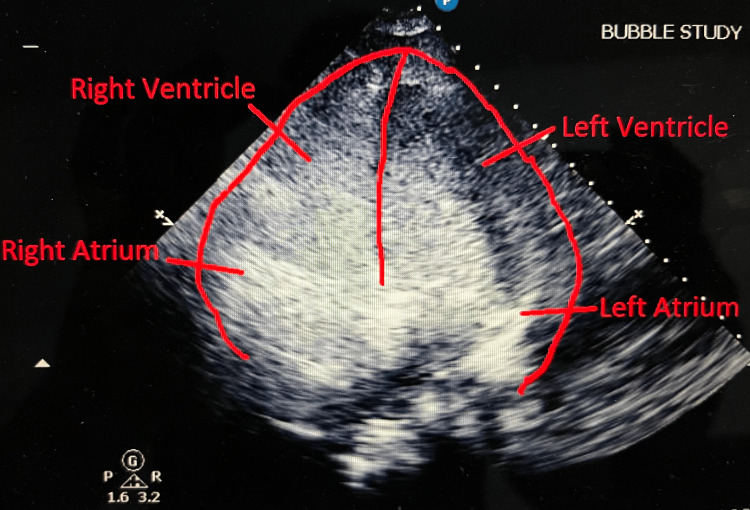
Bubble study showing significant right to left shunting across PFO, with complete opacification of the left ventricle at the P2 setting. PFO, patent foramen ovale

**Figure 3:**
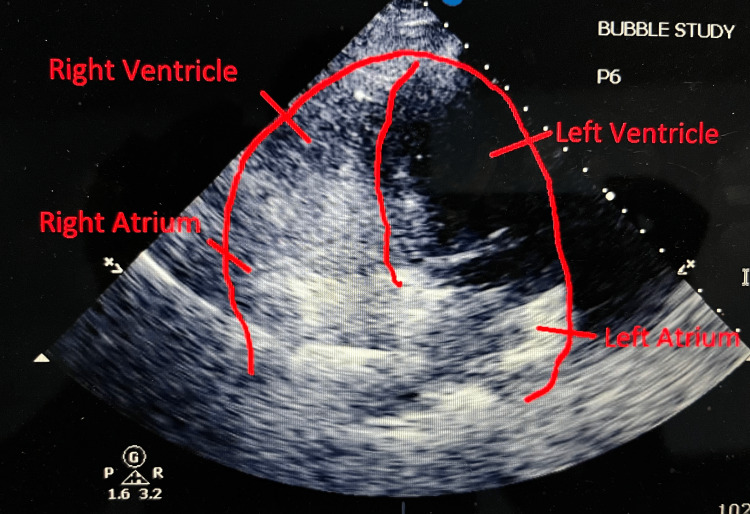
Bubble study showing a significant decrease in the right to left shunting across PFO at P6 setting/Impella RP support. PFO, patent foramen ovale

Right ventricular Impella RP was placed under fluoroscopic guidance for hemodynamic support, extending from the inferior vena cava to the pulmonary artery. The adequate flow was obtained at the P6 setting. Following the placement of Impella RP, her oxygenation improved drastically, FIO2 requirement decreased from 100% to 40%. Repeat echo with bubble study showed an RTLS. The first injection at the P2 setting showed significant RTLS across PFO, with complete opacification of the left ventricle (Figure 2). The second injection repeated at the P6 setting also showed a RTLS, but the density of bubbles in the left side of the heart was significantly less compared to the first injection indicating a decrease of the shunt on Impella support (Figure 3). Her hemodynamics also improved drastically after the Impella placements. Vasopressin and norepinephrine were discontinued, but dobutamine was continued for inotropic support.

She developed a fever with a temperature of 101°F. She received broad-spectrum antibiotics to cover for hospital-acquired infection. Her creatinine also trended up from baseline 1.3 to 5.4 mg/dL, likely due to cardiogenic shock resulting in pre-renal plus cardiorenal with the component of contrast-induced nephropathy. Nephrology was consulted, and the patient was started on continuous venovenous hemodialysis (CVVHD). She also developed thrombocytopenia that decreased from 300,000 to 50,000 per microliter. Her hemoglobin dropped with elevated lactate dehydrogenase (LDH), which looked like a consumptive etiology secondary to the device. She also developed cyanosis in her lower extremities which appeared to be limb ischemia, most likely due to her support devices. She also had worsening lactic acidosis on serial arterial blood gas (ABGs), with hyperkalemia and low bicarbonate. She received calcium gluconate, insulin, dextrose, and lokelma and was started on a bicarbonate drip. However, because of the need to continue support devices, IABP and Impella were continued.

The patient had multiple episodes of sustained ventricular tachycardia and bradycardia; she was shocked 25 times during that episode and received amiodarone, lidocaine, epinephrine, and atropine. She became pulseless, followed by multiple rounds of cardiopulmonary resuscitation (CPR), resulting in her unfortunate demise. 

## Discussion

Right ventricular infarction results from occlusion of RCA proximal to the acute marginal branches, which supply the RV. The decreased blood flow causes acute RV ischemic dysfunction with a decrease in the overall compliance of the wall, thereby resulting in elevated right ventricular pressure. The RV has a unique resilience property that helps complete recovery soon after the blood flow is re-established [5]. Right ventricular infarction can also lead to other complications like high-degree atrioventricular (AV) block, acute RV failure, and the opening of latent PFO [6]. If the occlusion of RCA is proximal to the right atrial branch, it can also cause ischemia of the right atrium with loss of right atrial kick. This further worsens the RAP resulting in the stretching of PFO with the eventual shunt. Although it is a rare complication, the development of refractory hypoxemia after RV myocardial infarction (MI) should always alert clinicians to consider the possibility of shunting across PFO. Our patient developed refractory hypoxemia requiring 100% oxygen via mechanical ventilation. No other causes of hypoxemia were identified, raising a strong suspicion for a RTLS. 

Right ventricular infarction is diagnosed by history, physical examinations, electrocardiogram (EKG) findings, elevated cardiac enzymes, echo, and confirmed with coronary angiography. EKG reveals ST/T wave changes in inferior and right-sided EKG leads [7]. The diagnosis of PFO is made with transthoracic or transesophageal echo with a bubble study, which shows the crossing of bubbles from the right atrium to the left atrium [8].

The initial management of refractory hypoxemia secondary to PFO includes RV support and treatment of MI. In most cases, reperfusion of occluded RCA leads to improvement of RV function and hemodynamics with improvement in hypoxemia [7]. But recovery can lag in some patients who require pharmacological and mechanical support. Pharmacological support includes inotropes and vasopressors like dobutamine, norepinephrine, epinephrine, and vasopressin. Mechanical interventions can be used if the hemodynamics do not improve with reperfusion or medical management [6]. IABP is used to unload the left ventricle with subsequent improvement in RV hemodynamics. In case of severe or refractory hypotension, the percutaneous RV assists devices like Impella RP which promptly improve hemodynamics and refractory hypoxemia. A subanalysis done on 14 patients showed favorable survival outcomes with Impella RP in RV predominant cardiogenic shock [9]. The use of Impella in RV failure complicated by PFO has been published in only one case report so far [4]. Our patient failed reperfusion after coronary angioplasty and developed severe and refractory hemodynamic compromise. The pharmacological interventions with inotropes and vasopressors were unsuccessful. She also had an IABP without improvement. Right heart catheterization showed elevated RAP and RV pressure. Impella RP placement improved the hemodynamics promptly with the rise in cardiac index, decrease in oxygen requirement from 100% to 40%, and successful discontinuation of vasopressors. 

Though there are not enough studies, it is believed that the transient shunt due to RV infarction decreases when the RV function recovers. As such, immediate closure of PFO is not warranted. However, permanent closure of the opening is considered if PFO persists [10].

## Conclusions

Right to left atrial shunt across a latent foramen ovale is a rare cause of refractory hypoxemia after RV infarction. Impella RP can be considered in such patients, which helps to lower the elevated right heart pressure and decrease the shunt, thereby providing a bridge to recovery.
